# Multicenter study of the efficacy and safety of electrocautery-enhanced lumen-apposing metal stents for the internal drainage of pancreatic fluid collections

**DOI:** 10.1038/s41598-024-53785-8

**Published:** 2024-03-06

**Authors:** Chen-Shuan Chung, Yu-Ting Kuo, Yi-Chun Chiu, Yang-Chao Lin, Chi-Ying Yang, Kuan-Chih Chen, Szu-Chia Liao, Cheuk-Kay Sun, Yen-Chih Lin, Hsiu-Po Wang

**Affiliations:** 1 Division of Gastroenterology and Hepatology, Department of Internal Medicine, Far Eastern Memorial Hospital, New Taipei City, Taiwan; 2 College of Medicine, Fu Jen Catholic University, New Taipei City, Taiwan; 3 Department of Internal Medicine, National Taiwan University Hospital, National Taiwan University College of Medicine, Taipei, Taiwan; 4 Division of Hepato-Gastroenterology, Department of Internal Medicine, Kaohsiung Chang Gung Memorial Hospital and Chang Gung University College of Medicine, Kaohsiung, Taiwan; 5 Department of Gastroenterology and Hepatology, Fu Jen Catholic University Hospital, New Taipei City, Taiwan; 6 Department of Internal Medicine, Digestive Medicine Center, China Medical University Hospital, Taichung, Taiwan; 7 Division of Gastroenterology and Hepatology, Department of Internal Medicine, Taichung Veterans General Hospital, Taichung, Taiwan; 8 Department of Internal Medicine, Shin Kong Wo Ho-Su Memorial Hospital, Taipei, Taiwan; 9 Department of Internal Medicine, Changhua Christian Hospital, Changhua, Taiwan

**Keywords:** Endoscopic ultrasound, Lumen-apposing metal stent, Pancreatitis, Pseudocyst, Walled-off necrosis, Therapeutic endoscopy, Pancreatitis

## Abstract

Pancreatic fluid collections (PFCs) including pancreatic pseudocyst (PP) and walled-off necrosis (WON) are complications after acute pancreatitis. We aimed to evaluate the efficacy and safety of endoscopic ultrasound (EUS)-guided lumen-apposing metal stent (LAMS) placement to manage PFCs. Between June 2019 and May 2023, patients with symptomatic PFCs who underwent EUS-guided electrocautery-enhanced LAMS drainage were enrolled retrospectively from eight tertiary centers in Taiwan. In total, 33 [14 (42.42%) PP and 19 (57.58%) WON] patients were enrolled. Gallstones (27.27%) and abdominal pain (72.73%) were the most common etiology and indication for drainage. The technical and clinical success rates were 100% and 96.97%, respectively, and the mean procedure time was 30.55 (± 16.17) min. Complications included one (3.03%) case of self-limited bleeding; there were no cases of mortality. Seven (21.21%) patients had recurrence. Patients with disconnected pancreatic duct syndrome (DPDS) had a higher recurrence rate than those without (71.43% vs. 38.46%, p = 0.05). After replacing LAMSs with transmural double-pigtail plastic stents (DPSs) in the DPDS patients, the DPS migration rate was higher in the patients with recurrence (100% vs. 33.33%, p = 0.04). In conclusion, drainage of symptomatic PFCs with EUS-guided electrocautery-enhanced LAMS appears to be efficient and safe. Replacing LAMSs with DPSs in DPDS patients was associated with a lower recurrence rate.

## Introduction

Pancreatic fluid collections (PFCs) are one of the most common complications of acute pancreatitis (AP). After 3–4 weeks of severe AP, some collections of fluid or necrotic tissue may be encapsulated outside the pancreas, which can lead to the formation of a pancreatic pseudocyst (PP) or walled-off necrosis (WON)^1^. Approximately 20–30% of patients with AP complicated with persistent PFCs have been reported to have PFC-related symptoms or complications, including fever, infection, abdominal pain, biliary or gastrointestinal tract obstruction, and aneurysm formation, for which interventions are required^2^. Step-up care for symptomatic PFCs includes cessation and control of the offending factors, accompanied with medical therapy such as proton pump inhibitors, somatostatin, pancreatic enzyme supplements, and antibiotics to control infection^2^. However, a substantial proportion of patients with persistent symptomatic PFCs fail conservative treatment and are referred for drainage of infective fluid or removal of necrotic tissues. Conventionally, drainage of PFCs is performed by either percutaneous or surgical methods. The former is performed by percutaneous pigtail drainage and the creation of a cutaneous fistula with a self-expandable metal stent followed by necrosectomy, while the latter includes either open or laparoscopic necrosectomy and video-assisted retroperitoneal debridement^2–5^. However, percutaneous interventions are associated with a low resolution rate and high re-intervention and recurrence rates, and surgical management is associated with high rates of perioperative mortality and comorbidities, fistula formation, longer hospital stay, and poor quality of life^5–7^.

Endoscopic therapy including transpapillary and transmural internal drainage is an alternative to conventional interventions. These endoscopic techniques have a higher clinical success rate compared with percutaneous drainage, and lower risks of multiorgan failure, fistula formation, and shorter hospital stay than surgery^5–7^. Traditional endoscopic internal drainage involves the creation of a transmural fistula followed by plastic stenting. However, only infective fluid can be drained into the gastrointestinal tract with plastic stents, and the necrotic tissue remains in the walled-off cavity, leading to persistent symptoms and recurrence. In 2012, Binmoeller et al.^8^ published a porcine study using a novel metal stent, the so-called lumen-apposing metal stent (LAMS), to create a communicating tract between two isolated organs. Subsequently, endoscopic ultrasound (EUS)-guided LAMS placement has been used in several off-label indications with high technical and success rates^9^. Transoral endoscopic necrosectomy for WON can be achieved with the use of LAMSs, which have been shown to be superior to plastic stents in terms of procedure time, need for surgery, and clinical success and recurrence rates^10^. However, most previous studies on the application of LAMSs for the management of PFCs have not used electrocautery enhancement, and thus the technique requires a fine needle puncture followed by guidewire insertion and tract dilatation^10–22^. In this multicenter study, we aimed to investigate the efficacy and safety of a novel electrocautery-enhanced LAMS technique which avoided the need for a preceding needle puncture and guidewire advancement for the management of symptomatic PFCs. We also evaluated the predictors of clinical resolution and recurrence.

## Methods

### Study design

**Figure 1:**
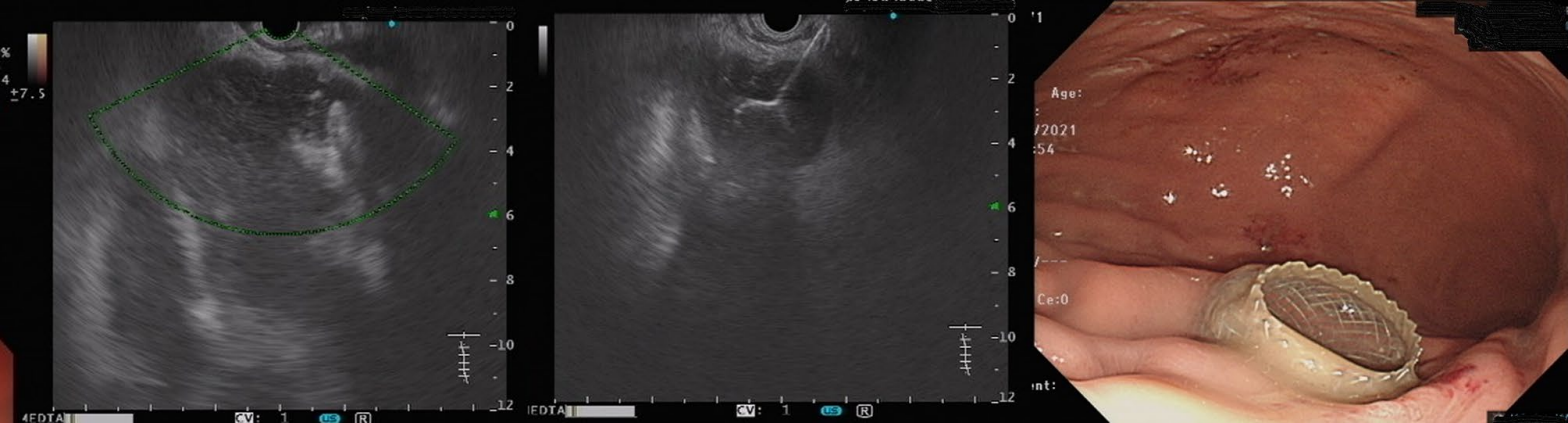
Endoscopic ultrasound (EUS)-guided deployment of a lumen-apposing metal stent (left & middle: EUS imaging; right: endoscopic imaging).

**Figure 2:**
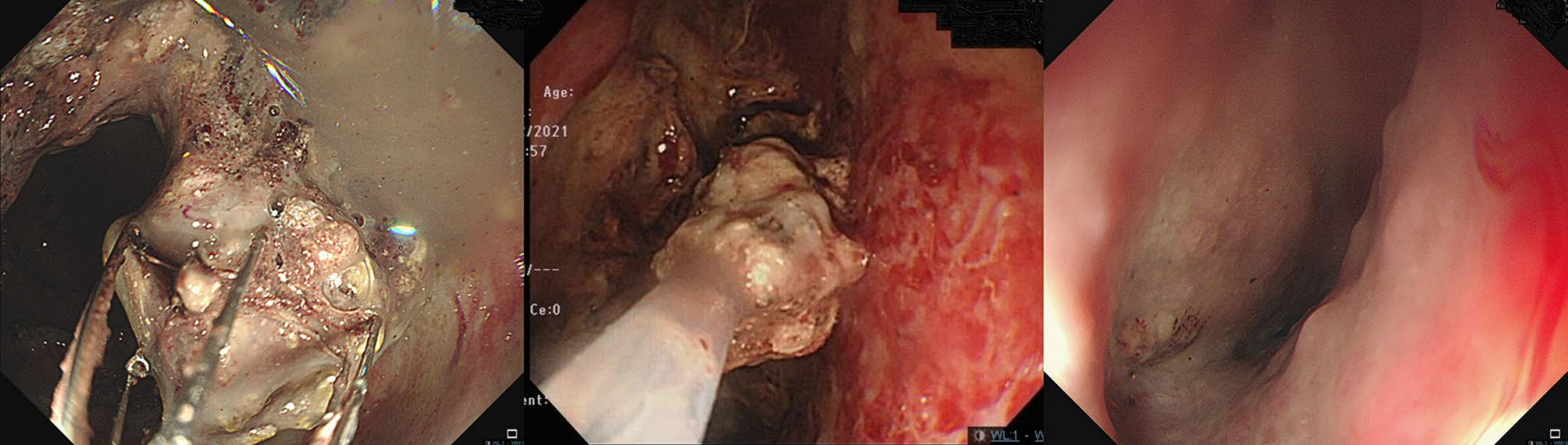
Direct endoscopic necrosectomy (left & middle: removal of necrotic tissue using a grasper; right: complete clearance of necrotic parts and resolution of walled-off necrosis).

**Figure 3:**
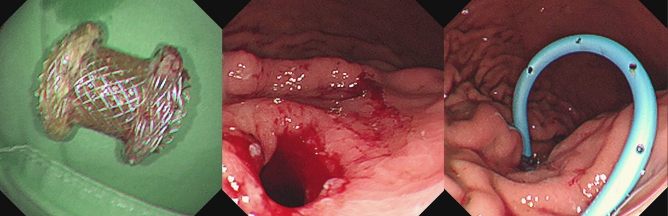
Removal of a lumen-apposing metal stent and replacement with a transmural double-pigtail plastic stent (left: removed metal stent; middle: fistula formation at cysto-gastrostomy; right: insertion of a double-pigtail plastic stent through cysto-gastrostomy).

**Figure 4:**
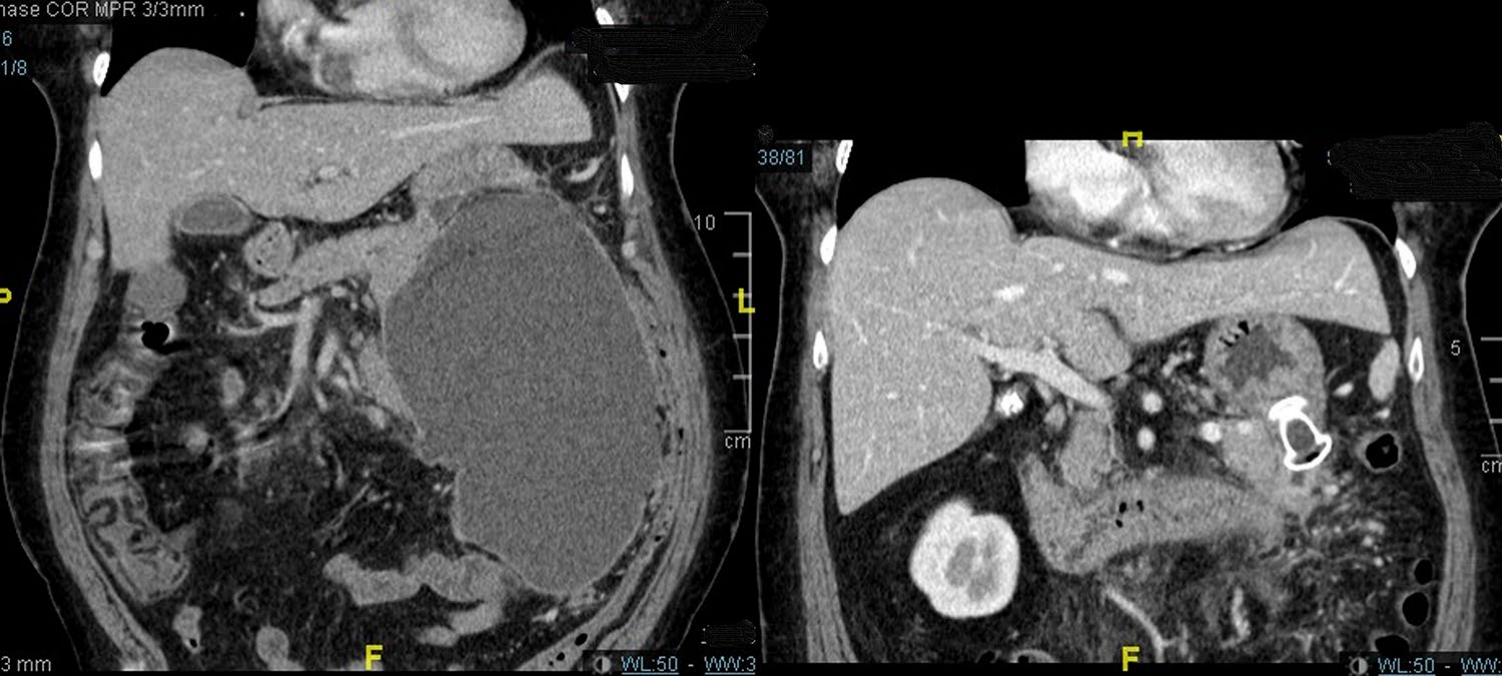
Computed tomography imaging of pancreatic walled-off necrosis before (left) and after (right) endoscopic ultrasound-guided lumen-apposing metal stent placement and direct endoscopic necrosectomy.

This retrospective multicenter study was conducted at eight tertiary centers in Taiwan, and it was approved by the Research Ethics Review Committees of the study institutes (Far Eastern Memorial Hospital FEMH-111171-E, China Medical University Hospital CMUH111-REC2-114, Kaohsiung Chang Gung Memorial Hospital 202201132B0D001, Changhua Christian Hospital CCH210103, Fu Jen Catholic University Hospital FJUH111205, and Shin Kong Wo Ho-Su Memorial Hospital 20221012R). All study methods were performed in accordance with the Committee on Publication Ethics guidelines. Codes rather than identifying information of the enrolled patients were used in this retrospective study, and the requirement for written informed consent was waived (Research Ethics Review Committee of Far Eastern Memorial Hospital). All data generated or analyzed during this study are included in this published article and its supplementary information files. Patients with AP who developed symptomatic PFCs and were treated by internal drainage with EUS-guided transmural LAMS placement (Figs. 1, 2, 3 and 4) between June 2019 and May 2023 were enrolled consecutively. All procedures were performed by endosonographers with more than 5 years of experience.

### Endoscopic ultrasound techniques

In this study, we used Hot AXIOS™ electrocautery-enhanced LAMSs (Boston Scientific, Marlborough, MA, USA) for internal drainage. The Hot AXIOS™ is a saddle-shaped braided flexible metal stent which is flanged at both ends and is fully covered by a silicon membrane. The sizes of the PFCs, defined as the proportion of necrotic area in WON, and the distance between the wall of the encapsulated PFC and gastrointestinal tract, were evaluated by EUS. Color Doppler was used to avoid intervening vessels at the puncture site and trajectory. Under EUS-guidance, an electrocautery-enhanced delivery system was deployed (autocut mode, 100 W, effect 5, VIO200D, ERBE, Germany) from the gastrointestinal lumen into the PFC, and then the bilateral flanges were deployed in order to approximate the PFC and gastrointestinal wall. After endoscopic and fluoroscopic confirmation of the iatrogenic communicating tract created by the LAMS, the delivery system was removed. If clinical indications were present, such as persistent PFC-related symptoms after LAMS placement, transoral direct endoscopic necrosectomy (DEN) using foreign body retrievers was performed through the LAMS to remove necrotic tissues in the pancreatic WON. The LAMS was removed if PFC-related clinical symptoms resolved, and double-pigtail plastic stents (DPSs) were inserted via cysto-gastroenterostomy at the discretion of the endoscopist. Once the diagnosis of disconnected pancreatic duct syndrome (DPDS) was confirmed, transmural DPSs were inserted via cysto-gastroenterotomy or transpapillary pancreatic stenting if possible after removal of LAMS.

### Outcome measurements

Clinical, radiological, and endoscopic data were reviewed from medical records. Technical success was defined as the ability to place and deploy a transmural LAMS and observe fluid gushing out under endoscopy after deployment during the index procedure. Clinical success was classified as symptom resolution, defined as disappearance of PFC-related symptoms within the first week of LAMS placement, and radiological resolution, defined as a drained PFC with a size less than 20 mm before LAMS removal. Adverse events (AEs) within 3 days were classified as immediate procedure-related events, and those occurring from 3 days until LAMS removal as delayed complications. DPDS was diagnosed if a disrupted pancreatic duct was shown in EUS, endoscopic retrograde pancreatography, or cross-sectional imaging studies including computed tomography and magnetic resonance imaging. Long-term clinical success was defined as complete resolution of the PFC-related symptoms at the last follow-up date. Recurrence was defined as the development of PFC-related symptoms or when the size of the PFC increased after complete resolution with the initial EUS-guided LAMS management.

### Statistical analysis

The Student’s t-test and χ^2^ test or Fisher’s exact test were used to compare continuous and categorical variables, respectively. Continuous variables were expressed as mean (± standard deviation), and categorical variables were expressed as count (%). Retrospective analysis of consecutive patients with PFCs who underwent EUS-guided electrocautery-enhanced LAMS placement was conducted without sample size estimation. We divided the patients into two groups according to PFC recurrence, and univariate logistic regression analysis was used to evaluate the predictors, which were expressed as odds ratio (OR) for outcome assessment. Statistical significance was defined as a two-tailed p value < 0.05. The statistical analysis was performed using STATA software (version 11.0; Stata Corp, College Station, TX, USA). All data generated or analyzed during this study are included in this published article and its supplementary information files.

## Results

### Characteristics of the enrolled patients and PFCs

The demographic data of the enrolled patients are shown in Table 1. Thirty-three patients (13 females; 20 males; mean age 53.93 ± 15.28 years) with symptomatic PFCs were enrolled. Only three (9.09%) patients did not have comorbidities, while six (18.18%) patients had malignancy (one ovarian cancer, one endometrial cancer, one pancreatic ductal adenocarcinoma, one intraductal papillary mucinous neoplasm and two pancreatic neuroendocrine tumors). The most common etiology of AP was gallstones (27.27%), followed by alcohol (24.24%), hypertriglyceridemia (12.12%), malignancy (15.15%), and others (21.21%, two idiopathic, two drug-related pancreatitis, one trauma, one post-endoscopic retrograde pancreatography and one post-endoscopic papillectomy). Abdominal pain was the most common (72.73%) reason for the intervention, followed by infection (21.02%) and gastric outlet obstruction (6.06%). Fourteen (42.42%) patients had a PP, and nineteen (57.58%) had WON. The mean PFC size was 10.77 (± 5.48) cm, and the mean necrotic area in WON was 63.50% (± 23.46%). Most PFCs were unilocular (75.76%), with Balthazar grades of C (15.15%), D (57.58%) and E (27.27%). Seven (21.21%) patients had paracolic gutter extension of inflammatory fluid collection. The mean follow-up period was 421.97 (± 397.75) days.

**Table 1:** Demographic data of the enrolled subjects and characteristics of the PFCs.

Variables	Patients with symptomatic PFCs (n = 33)
Age, mean ± SD (range), years	53.93 ± 15.28 (20–87)
Sex, female/male, n (%)	13 (39.39)/20 (60.61)
BMI, mean ± SD, kg/m^2^ (range)	24.13 ± 4.99 (17.00–37.66)
Comorbidities, n (%)	
None	3 (9.09)
Cardiovascular disease	6 (18.18)
Cerebrovascular disease	3 (9.09)
Chronic liver disease	5 (15.15)
Chronic kidney disease	2 (6.06)
Hypertension/DM/Hyperlipidemia	23 (69.69)
Malignancy	6 (18.18)
Etiology of pancreatitis, n (%)	
Gallstones	9 (27.27)
Alcohol	8 (24.24)
Hypertriglyceridemia	4 (12.12)
Malignancy*	5 (15.15)
Others^#^	7 (21.21)
Symptoms of the PFC, n (%)	
Abdominal pain	24 (72.73)
Infection	7 (21.21)
GI tract obstruction	2 (6.06)
Types of PFC, n (%)	
Pseudocyst	14 (42.42)
Walled-off necrosis	19 (57.58)
Proportion of necrotic area, mean ± SD (range), %	63.50 ± 23.46 (30.00–90.00)
Size of the PFC, mean ± SD (range), cm	10.77 ± 5.48 (3.30–25.00)
Characteristics of the PFC, n (%)	
CT Balthazar grade (A/B/C/D/E)	0 (0)/0 (0)/5 (15.15)/19 (57.58)/9 (27.27)
Unilocular/Multilocular (communicating/non-communicating)	25 (75.76)/8 (24.24) [4 (50.00)/4 (50.00)]
Paracolic gutter extension	7 (21.21)
Follow-up period, mean ± SD (range), days	421.97 ± 397.75 (65–2065)

BMI, body mass index; CT, computed tomography; DM, diabetes mellitus; GI, gastrointestinal; PFC, pancreatic fluid collection.

*Patients with malignancy: ovarian cancer (n = 1), intraductal papillary mucinous neoplasm (n = 1), pancreatic ductal adenocarcinoma (n = 1), pancreatic neuroendocrine tumor (n = 2).

^#^Other etiologies of pancreatitis: idiopathic (n = 2), drug-related pancreatitis (n = 2), post-endoscopic retrograde pancreatography (n = 1), post-papillectomy (n = 1), trauma (n = 1).

### Efficacy and safety of EUS-guided LAMS placement

Eight (24.24%), 10 (30.30%) and 15 (45.45%) patients underwent EUS-LAMS placement within 1 month, between 1 and 3 months, and more than 3 months after the onset of AP, respectively (Table 2). Most (66.67%) of the patients had not received an intervention before EUS-LAMS placement, while seven (21.21%) patients had undergone external pigtail drainage, two (6.06%) EUS-guided transmural DPS internal drainage, and two (6.06%) transpapillary pancreatic stenting (TPS) before EUS-LAMS placement. The LAMS size was 15 mm in all patients. The total procedure and puncture-to-deployment times were 30.5 (± 16.17) minutes and 5.76 (± 7.75) minutes, respectively. The most common transluminal route was from the gastric body (75.76%), followed by the gastric antrum (6.06%) and gastric fundus (6.06%). The technical success and symptom resolution rates were both 100%, and the radiological resolution rate was 96.97%. One (3.03%) patient had an early AE of procedure-related intracystic bleeding at 8 h after the procedure despite withholding clopidogrel for 7 days preoperatively. Fortunately, the bleeding resolved after conservative treatment with a blood transfusion and intravenous tranexamic acid. Among the patients with WON, a mean 2.65 (± 1.98) DEN sessions were performed. Complications during DEN included three (16.67%) cases of self-limited bleeding, two (11.11%) of stent migration, and one (5.56%) of symptomatic pneumoperitoneum. The mean indwelling LAMS time was 25.27 (± 10.09) days. Seven (21.21%) patients developed PFC recurrence, of whom two (28.57%) were asymptomatic and the others needed further interventions. The mean recurrence to removal of the LAMS time was 200.29 (± 184.11) days.

**Table 2:** Details of the EUS-guided LAMS placement procedures.

Variables	Patients with symptomatic PFCs (n = 33)
Time from onset of AP to LAMS placement, mean ± SD (range), days	166.50 ± 306.42 (18–1284)
≤ 1 month, n (%)	8 (24.24)
1–3 months, n (%)	10 (30.30)
> 3 months, n (%)	15 (45.45)
Previous intervention before LAMS placement, n (%)	
No treatment	22 (66.67)
External drainage	7 (21.21)
EUS-guided transmural DPS	2 (6.06)
Transpapillary pancreatic stent	2 (6.06)
Surgery	0 (0)
Size of the LAMS, n (%)	
10 mm	0 (0)
15 mm	33 (100)
20 mm	0 (0)
Procedure time, mean ± SD (range), minutes	
Total procedure	30.55 ± 16.17 (13–72)
Puncture-to-deployment	5.76 ± 7.75 (2–45)
Transmural route, n (%)	
Transgastric	
Antrum	2 (6.06)
Body	28 (75.76)
Fundus	2 (6.06)
Transduodenal	1 (3.03)
Technical success, n (%)	33 (100)
Clinical success, n (%)	
Symptoms resolved	33 (100)
Radiologically resolved	32 (96.97)
Symptoms and radiologically resolved	32 (96.97)
Adjunct therapy, n (%)	
Concurrent external drainage	6 (18.18)
Concurrent transmural DPS	2 (6.06)
Hydrogen peroxide instillation	2 (6.06)
Nasocystic tube	0 (0)
Index procedure-related complications, n (%)	
Bleeding	1 (3.03)
Cyst rupture	0 (0)
Infection	0 (0)
Stent migration	0 (0)
Mortality	0 (0)
Others	0 (0)
DEN session, mean ± SD (range), times	2.65 ± 1.98 (0–7)
Accessories used for DEN, n (%)	
Patients underwent DEN	18 (54.55)
Pentapod grasper alone	9/18 (50.00)
Combination	9/18 (50.00)
DEN-related complications, n (%)	
Self-limited bleeding	3/18 (16.67)
Stent migration	2/18 (11.11)
Pneumoperitoneum	1/18 (5.56)
LAMS indwelling time, mean ± SD (range), days	25.27 ± 10.09 (3 –44)
Recurrence, n (%)	7/33 (21.21)
Asymptomatic without treatment	2/7 (28.57)
EUS-DPS	2/7 (28.57)
Use of antibiotics	1/7 (14.29)
External drainage	1/7 (14.29)
Surgery	1/7 (14.29)
EUS-LAMS	0 (0)
Recurrence time after LAMS removal, mean ± SD (range), days	200.29 ± 184.11 (28–542)

AP, acute pancreatitis; DEN, direct endoscopic necrosectomy; DPS, double-pigtail stent; EUS, endoscopic ultrasound; LAMS, lumen-apposing metal stent; PFC, pancreatic fluid collection.

### Predictors of recurrence

There were no significant differences in the etiology of pancreatitis, characteristics of the PFCs, and AP onset to LAMS placement time between the patients with and without PFC recurrence (Table 3). However, more of the patients with recurrence had DPDS (71.43% vs. 38.46%, p = 0.047). In addition, the patients with DPDS who developed recurrence had a higher migration rate of the replacement transmural DPS after LAMS removal compared to those with resolution (100% vs. 33.33%, p = 0.038). Univariate analysis showed that older age (OR 1.02), male sex (OR 1.83), WON type (OR 6.00), paracolic gutter extension (OR 4.13), more sessions of DEN (OR 1.38), presence of DPDS (OR 7.50), and those in whom the LAMS was not replaced with a transmural DPS (OR 2.13) were associated with a higher risk of PFC recurrence, although all without statistical significance (Table 4).

**Table 3:** Clinical outcomes of the enrolled subjects.

Variables	Resolution (n = 26)	Recurrence (n = 7)	P value
Etiology of pancreatitis, n (%)			
Gallstones	5 (19.23)	4 (57.14)	
Alcohol	7 (26.92)	1 (14.29)	
Hypertriglyceridemia	3 (11.54)	1 (14.29)	
Malignancy	5 (19.23)	0 (0)	
Others	6 (23.08)	1 (14.29)	0.104
Characteristics of the PFC, n (%)			
CT Balthazar grade (C/D/E)	5 (19.23)/14 (53.85)/7 (26.92)	0 (0)/5 (71.42)/2 (28.57)	0.487
Unilocular/Multilocular	18 (69.23)/8 (30.77)	7 (100.00)/0 (0)	0.092
Communicating/Non-communicating	22 (84.62)/4 (15.38)	7 (100)/0 (0)	0.268
Type of PFC, PP/WON, n (%)	13 (50.00)/13 (50.00)	1 (14.29)/6 (85.71)	0.045
Size of the PFC, mean ± SD, cm	10.61 ± 5.89	11.36 ± 3.84	0.755
Time of onset to LAMS placement, mean ± SD, days	145.42 ± 247.12	256.86 ± 455.96	0.388
Less than 1 month, n (%)	7 (26.92)	1 (14.29)	
More than 1 months, n (%)	19 (73.08)	6 (57.14)	0.488
Presence of DPDS, n (%)	10 (38.46)	5 (71.43)	0.047

Variables	Patients with DPDS and resolution (n = 10)	Patients with DPDS and recurrence (n = 5)	P value
Transmural DPS after LAMS removal, n (%)	6 (60.00)	4 (80.00)	0.438
Migration	2/6 (33.33)	4/4 (100.00)	0.038
Transpapillary pancreatic stent after LAMS removal, n (%)	6 (60.00)	2 (40.00)	0.575
Migration	4/6 (66.67)	0/2 (0.00)	0.103
Concurrent transpapillary pancreatic stent and transmural DPS after LAMS removal, n (%)	4 (40.00)	2 (40.00)	1.000

DPDS, disconnected pancreatic duct syndrome; DPS, double-pigtail stent; LAMS, lumen-apposing metal stent; PFC, pancreatic fluid collection; PP, pancreatic pseudocyst; WON, walled-off necrosis.

**Table 4:** Univariate analysis for the predictors of PFC recurrence.

	Odds ratio	95% confidence interval	P value
Age (every 1 year)	1.02	0.96–1.08	0.565
Male sex	1.83	0.30–11.26	0.513
PFC size (every 1 cm)	1.02	0.88–1.19	0.746
PFC type (WON vs. PP)	6.00	0.63–57.06	0.119
WON necrotic area	1.00	0.96–1.05	0.848
Paracolic gutter extension	4.13	0.66–25.90	0.131
DEN session	1.38	0.83–2.30	0.219
Presence of DPDS	7.50	0.76–74.16	0.085
Without transmural DPS after LAMS removal	2.13	0.39–11.59	0.380

DEN, direct endoscopic necrosectomy; DPDS, disconnected pancreatic duct syndrome; DPS, double-pigtail stent; LAMS, lumen-apposing metal stent; PFC, pancreatic fluid collection; PP, pancreatic pseudocyst; WON, walled-off necrosis.

## Discussion

Pancreatic fluid collections are one of the most common complications after severe AP^1^. Traditional approaches for symptomatic PFCs such as percutaneous and surgical interventions are often challenging, with high re-intervention and recurrence rates, peri-operative comorbidities, and mortality^4,5,7^. With advances in interventional EUS techniques, endoscopic treatment has replaced invasive procedures in the treatment of several biliopancreatic disorders^13^. To our knowledge, the present study is the first to demonstrate the efficacy and safety of managing symptomatic PFCs after severe AP using EUS-guided internal drainage with an electrocautery-enhanced rather than conventional LAMS system in Taiwan. Most previous studies on the application of LAMSs for the management of PFCs have not used an electrocautery-enhanced technique^10,12–22^. Therefore, tract creation using a fine needle with guidewire advancement and either mechanical or electrocautery-assisted dilatation is necessary. This requires a longer procedure time, and technical failure can occasionally occur when exchanging accessories. In our study, we used freehand placement of the LAMSs with an electrocautery-enhanced tip, and this technique did not require a fine needle puncture, guidewire insertion or dilatation for tract creation. This may have shortened the procedure time and resulted in the high technical (100%) and clinical (96.97%) success rates, with low complication rate (3.03%). The presence of DPDS was associated with recurrence, and we suggest replacing the LAMSs with a transmural DPSs in patients with DPDS to prevent symptomatic PFC recurrence.

Peripancreatic fluid with interstitial edema can occur in patients with severe AP, and transient organ failure and mortality have been reported in the first 2 weeks after onset^2^. The encapsulated collection of fluid or necrotic tissue has been reported in approximately 6–20% of patients with AP and 20–40% of those with chronic pancreatitis after 4 weeks of the disease, and cross-sectional imaging may disclose well-circumscribed intra- or extra-pancreatic homogeneous fluid density or non-liquid density, with varying degrees of loculation^1–3,14–16^. Most patients with chronic PFCs have spontaneous regression and do not require an intervention, however 15 and 30% of patients with PP or WON develop symptoms or complications, such as abdominal pain, infection, and biliary or enteral obstruction^2,3^. Surgical interventions or percutaneous drainage are the traditional standards of care for symptomatic PFCs, however complication and mortality rates of 64–95% and 6.7–64.1%, respectively, have been reported in patients undergoing a surgical intervention for PFCs^4^. A meta-analysis of 190 patients reported significantly lower risks of new-onset multiorgan failure (OR 0.31, 95% CI 0.10–0.98), perforations of visceral organs or enterocutaneous fistulae (OR 0.31, 95% CI 0.10–0.93), and pancreatic fistulae (OR 0.09, 95% CI 0.03–0.28) as well as a shorter hospital stay in patients who received endoscopic treatment compared to those who underwent surgery^6^. Another meta-analysis of six studies comparing endoscopic and percutaneous drainage of PFCs reported a higher re-intervention rate and lower resolution rate among patients receiving percutaneous therapy^7^. Therefore, a minimally invasive procedure with an endoscopic approach seems to be superior to conventional surgical and percutaneous methods in terms of clinical succuss, complications, recurrence, and quality of life.

Several modalities of endoscopic therapy are available for PFCs, including EUS-guided transmural cystoenterostomy for internal drainage, transoral endoscopic necrosectomy, and TPS^11^. Binmoeller et al.^8^ published a landmark report in which they used a novel lumen-apposing stent to create a gastroenterostomy and facilitate intubation with a gastroscope for further endoscopic treatment in a porcine model. Several specialized LAMSs and delivery devices have subsequently been introduced to provide endoscopists with an alternative to surgical interventions. LAMSs were initially approved in 2013 for the drainage of PP and WON with less than 30% solid debris, and thereafter their use has been expanded to many off-label indications, including gastroenterostomy, biliary and gallbladder drainage, and temporary gastric access for endoscopy^9^. Several clinical studies have demonstrated that EUS-guided internal drainage of symptomatic PFCs with LAMSs is an efficient and safe procedure, with technical and clinical success rates of 91–100% and 79–98%, respectively, and a major AE rate of less than 5%^12–18^. Compared with EUS-guided internal drainage using plastic stents, LAMSs have been associated with a higher clinical success rate (88–100% vs. 80–92%), shorter procedure time (10.5–14.9 min vs. 21.4–63.6 min), and lower recurrence rate (6.3–40% vs. 18.8–41.7%) in many clinical studies^19–23^. A multicenter retrospective study involving 14 institutes and 189 patients found that EUS-guided transmural drainage of WON with LAMSs had a higher clinical success rate (80.4% vs. 57.5%, p = 0.001), shorter procedure time (50.4 min vs. 64.6 min, p = 0.003), lower need for surgery (5.6% vs. 16.1%, p = 0.023), and lower recurrence rate (5.6% vs. 22.9%, p = 0.036) than plastic stents^10^. When performing DEN for WON, it is sometimes necessary to use multiple plastic stents followed by balloon dilation to create a larger tract for the gastroscope. In contrast, the saddle part of a LAMS has a larger diameter, which provides access for necrosectomy by intubating the transoral gastroscope directly into the PFC. LAMSs have been reported to have a higher DEN success rate compared to plastic stents (80.4% vs. 57.5%, p = 0.001)^10^. Taken together, these findings suggest that LAMSs can achieve more rapid control of infection and symptoms with less recurrence than plastic stents. In this study, we placed LAMSs with an electrocautery-enhanced tip using a freehand technique without first creating a tract. The technical and clinical success rates were 100% and 96.97%, respectively, which are higher than those using conventional LAMSs without electrocautery-enhanced tips reported in the literature (97.9–100% and 80.4–96%)^9,10,12,14,16^. In addition, the overall AE rate (3.03%) was lower than that reported for LAMSs without electrocautery-enhanced tips (9.8–24.3%)^9,10,12,14,16^. Only one patient developed self-limited postprocedural bleeding in our study.

The recurrence of PFCs and clinical success after EUS-guided drainage have been associated with the proportion of necrotic area in WON and the presence of DPDS. Maringhini et al.^24^ reported that more than 60% of their patients with a solid necrotic area of over 50% required more than three DEN sessions. In addition, the clinical success rate of EUS-guided drainage has been reported to be lower in patients with WON and a necrotic area of more than 40% (OR 0.10, 95% CI 0.02–0.60, p = 0.01)^25^. Several adjunct methods of DEN for WON have been reported in the literature, including using local instillation or nasocystic tube irrigation with normal saline, hydrogen peroxide, streptokinase or antibiotics^26,27^. However, none of them are currently recommended as an appropriate technique for DEN due to limited evidence^28^. After the resolution of PP and complete clearance of necrotic parts in WON with a collapsed cavity, the LAMS should be removed as soon as possible to prevent delayed complications. Regarding the timing of LAMS removal, an expert panel recommended removal at a mean time of 4.59 weeks^7^. However, the presence of DPDS, which is associated with a higher recurrence rate, should be investigated before LAMS removal. A retrospective review reported that 48 (50%) (6 PP and 42 WON) of 96 PFC patients had DPDS, and that those in whom DPSs were replaced with LAMSs had a lower recurrence rate (5% vs. 37%, p = 0.011)^29^. In addition, TPS was found to be associated with a higher successful clinical outcome rate in patients with DPDS (76.5% vs. 22.2%, p = 0.014) in a prospective study of 31 patients with DPDS^30^. Among patients with DPDS in our study, we have found that PFC recurrence was statistically significant higher in those with migration of transmural DPS and numerically higher in those with migration of transpapillary pancreatic stents after LAMS removal (Table 3). Thus, in patients with DPDS, long-term indwelling of transmural DPSs may be necessary to prevent recurrence, and transpapillary bridging of the disrupted main pancreatic duct, if possible, is recommended before removal of the transmural stent.

There are several limitations to this study. First, it was a retrospective study with a relatively small sample size and heterogeneous data, and we failed to identify independent risk factors for PFC recurrence in multivariate analysis. Because of reimbursement issues with the costly accessories associated with LAMSs in Taiwan, the number of candidate patients was limited. Therefore, we conducted this study at multiple centers, and further studies on the cost-effectiveness compared with other treatment modalities are warranted. Second, the practices used for the management of symptomatic PFCs were unique to each institute. Hence, our findings may not be generalizable to all centers. Third, not all of the enrolled patients underwent investigations for the presence of DPDS before LAMS removal and replacement with transmural DPSs. In addition, more than half (57.58%) of the enrolled patients with PFCs had WON with a mean necrotic area of 63.50%. Thus, the recurrence rate (21.21%) was higher than that reported in the literature.

In conclusion, EUS-guided internal drainage and endoscopic therapy via electrocautery-enhanced LAMSs for symptomatic PFCs appears to be an efficient and safe procedure. Before LAMS removal, investigations for the presence of DPDS and replacement with transmural DPSs are important to avoid recurrence. Further well-designed studies are warranted to compare this method with others and elucidate the appropriate indwelling times of the LAMSs and replacement plastic stents.

### Supplementary Information

Supplementary Information.

## Data Availability

All data generated or analyzed during this study are included in this published article and its supplementary information files. Data supporting this study are not publicly available and please contact chungchenshuan_3@yahoo.com.tw to access the original data.
